# Reporting quality of randomized trials in the diet and exercise literature for weight loss

**DOI:** 10.1186/1471-2288-5-9

**Published:** 2005-02-23

**Authors:** Cheryl A Gibson, Erik P Kirk, James D LeCheminant, Bruce W Bailey, Guoyuan Huang, Joseph E Donnelly

**Affiliations:** 1 Department of Internal Medicine, University of Kansas School of Medicine, Kansas City, KS, 66160, USA; 2 Center for Human Nutrition, Washington University in Saint Louis School of Medicine St. Louis, Missouri, 63110, USA; 3 Energy Balance Laboratory & The Center for Physical Activity and Weight Management The Schiefelbusch Institute for Lifespan Studies University of Kansas Lawrence, KS, 66045, USA; 4 Department of Physical Education, University of Southern Indiana, Evansville, IN, 47712, USA

## Abstract

**Background:**

To adequately assess individual studies and synthesize quantitative research on weight loss studies, transparent reporting of data is required. The authors examined the reporting quality of randomized trials in the weight loss literature, focusing exclusively on subject characteristics as they relate to enrollment, allocation, and follow-up.

**Methods:**

An extensive literature review, which included a computerized search of the MEDLINE database, manual searches of bibliographic references, and cross-referencing of 92 review articles was conducted. A checklist, based on CONSORT recommendations, was used to collect information on whether or not authors reported age, gender, co-morbid disease, medication use, race/ethnicity, and postmenopausal status. Also tracked was whether or not initial and final sample size was reported and stratified by gender.

**Results:**

Of 604 possible articles, 231 articles met eligibility criteria. Important subject characteristics were not reported as the following breakdown indicates: age (11%), gender (4%), race/ethnicity (86%), co-morbid disease states (34%), and medication use (92%). Additionally, 21% of articles failed to report initial sample size by gender while 69% neglected to report final sample size by gender.

**Conclusion:**

Inadequate reporting can create difficulties with interpretation and can lead to biased results receiving false credibility. The quality of reporting for weight loss studies needs considerable improvement.

## Background

Current statistics indicate that approximately 64.5% of US adults can be considered overweight while 30.5% can be classified as obese [1]. Obesity is also linked to a variety of chronic diseases, and is associated with approximately 300,000 deaths each year and annual economic costs of over $117 billion [2]. According to the latest Behavioral Risk Factor Surveillance Survey (BRFSS) data, obesity and overweight continue to be major pubic health concerns, and reports indicate that no state had met the Healthy People 2010 objective of reducing obesity to 15% [3]. As a result, there is need for a strong evidence base for prevention and treatment strategies.

Systematic reviews can offer the most reliable sources of information on which to develop guidelines and base treatment policy. However, such reviews need to contain a high proportion of all relevant evidence, which relates both to the need to find all trials and the need to analyze data on all participants [4]. To improve the quality of reporting for randomized controlled trials (RCTs) in the overweight and obesity literature, as well as any intervention trial, investigators are encouraged to publish reports in clear and unambiguous language, accounting for all events that occurred during the conduct of the investigation, and providing an accurate and thorough description of subjects who were selected, excluded, withdrawn or did not complete the study. Failing to report essential variables, such as subject characteristics, does not allow readers to adequately judge the validity or applicability of the study results, and may influence the interpretation of findings [5].

In response to the need for quality reporting, a panel of clinical investigators, epidemiologists, biostatisticians, and journal editors published a statement called the Consolidation of the Standards of Reporting Trials (CONSORT), which is designed to improve the standard of written reports of RCTs [6]. The CONSORT statement includes a checklist of 21 items and a flow diagram that can be used by authors to mark the page of the manuscript in which each of the items is addressed. In addition, the flow chart provides a detailed description of the progression of subjects through the intervention trial, from the number of potentially eligible participants for inclusion in the study to the number of intervention subjects in each group who completed the trial [6]. Using a similar format, we adapted the CONSORT form to examine the frequency of explicit reporting of subject characteristics as they relate to enrollment, allocation, and follow-up for weight loss studies published in the diet and exercise literature.

## Methods

For the present study, we used information collected from a meta-analysis of the diet and exercise literature. The primary purpose of the meta-analysis was to statistically integrate and analyze published research studies on the effects of diet restriction only, diet restriction and exercise, or exercise only on weight loss, body composition, fat distribution, metabolism, and aerobic fitness. We examined the frequency of explicit reporting of subject characteristics (i.e., age, gender, co-morbid disease states, medication use (in addition to intervention drug), race/ethnicity, and postmenopausal status). We also examined whether or not initial and final sample size was reported and if so, we recorded if the final sample size was reported by gender.

Search strategy. With the assistance of a clinical medical librarian, we developed search strategies, key words, and check tags to begin our initial search for relevant articles to include in the meta-analysis. We identified studies from a computerized search of the MEDLINE database (US National Library of Medicine) from 1966 – 2003, manual searches of bibliographic references of relevant published articles, and extensive cross-referencing and manual searches of 92 review articles.

### Overall study inclusion criteria

Subject types. We required that intervention trials must study overweight or obese (BMI ≥ 25 kg/m^2^) adult participants (≥ 18 years old). Ambulatory patients who were kept on wards exclusively for study purposes were also included. However, studies including pregnant women, patients with serious medical conditions, military personnel, and trained or professional athletes were excluded.

Intervention types. For inclusion, trials were required to meet each of the following criteria: 1) active intervention trials with one group assigned to a weight-loss program involving energy restriction, exercise (aerobic or weight training) or both energy restriction and exercise; 2) outcome measures included weight loss; however, weight loss was not required to be a primary outcome; 3) the minimum duration of the intervention for weight loss must be greater than one day; and, 4) the article must be published in the English language. Case studies, crossover trials (due to possible carryover effects of weight reduction), and exploratory studies were excluded. We did not require studies to have been conducted in the United States. Pharmacotherapy, hormonal therapy, or surgical treatment studies, which did not include a separate group receiving a weight loss intervention (i.e., diet and/or exercise) were not included in the review process.

Initial screening process. We independently screened all records resulting from our MEDLINE search strategies and manual searches by examining the titles and abstracts. We rejected articles if we could determine from the title and/or abstract that the study did not meet our selection criteria. If we could not determine whether or not to reject the article or if there was disagreement among reviewers, we retrieved the full text of the article for further evaluation.

### Quality control measures

To control for abstraction bias, we trained five graduate research assistants to extract information from articles, using sample articles. Reliability checks were completed on the first several sample articles before graduate students were allowed to code independently. On a monthly basis thereafter, inter- and intra-rater reliability checks were completed to control for drift. All research assistants were required to attend weekly meetings with the principal investigator and co-investigators to discuss coding issues and to resolve coding disagreements.

To control for potential selection biases after our initial screening procedure, we selected or rejected studies based on an examination of the research design and methodology. Acceptance or rejection for inclusion in the meta-analysis was not based on the study's outcome. We required coders to list specific reasons for all excluded studies. In addition, we tried to enhance data reporting for studies with missing covariate data by contacting authors by email or letter.

Data entry quality control measures included the design of screens to match the coding sheet, automatic range limits placed on items entered, and queries to check for outliers. Values greater than two standard deviations from the mean were checked against the article. In addition, a random sampling of 58 articles (~25%) was pulled to check for accuracy.

Standardized coding sheet for data abstraction. A separate checklist was devised based on CONSORT recommendations and quality indicators suggested by other authors [6-10]. Specific subject information was extracted from the meta-analysis database to examine if investigators reported age, race/ethnicity, and gender of their study subjects. Coders also recorded whether or not investigators reported the subjects' health status and presence of co-morbidities (i.e., diabetes, cardiac problems, cancer, or hypertension). Whether or not investigators reported medication use by subjects, other than the intervention drug, was also recorded. In addition, we tracked reporting of the postmenopausal status of subjects in investigations which included women 45 years or older. When analyzing the reporting quality for initial and final sample size separated by gender, only studies that included a group(s) consisting of both men and women were selected.

### Statistical analysis

The frequency of reporting in the journal articles for the selected variables was obtained by performing a proc frequency using SAS (Version 8.2, Cary, NC). Results are presented as totals.

## Results

**Figure 1:**
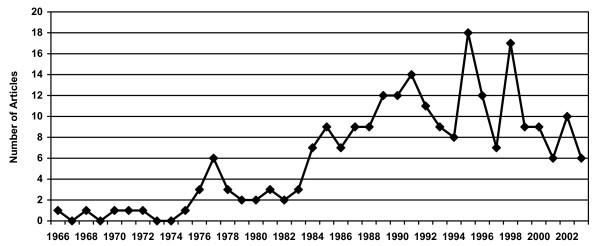
Frequency of randomized trials published by year.

Of the 604 randomized clinical trial articles that were reviewed, 231 (38%) met eligibility criteria for the present study. Figure 1 displays the frequency of articles included in the analysis by year of publication. Of these, 123 (53%) articles were randomized studies without a control group and 108 (47%) were randomized controlled trials. Seventy-six articles (33%) were dietary intervention only, 60 (26%) were exercise intervention only, and the remaining 95 articles (41%) included both exercise and dietary intervention for weight loss.

### Contribution of journals

For this analysis, the American Journal of Clinical Nutrition contributed the most articles that met the eligibility criteria (47), followed by the International Journal of Obesity (32), Medicine & Science in Sports & Exercise (24), Journal of the American Medical Association (11), Metabolism (11), New England Journal of Medicine (7), Archives of Internal Medicine (6), and Journal of the American Dietetics Association (6). In addition, 52 journals had 5 or fewer articles in this analysis.

### Subject characteristics

**Figure 2:**
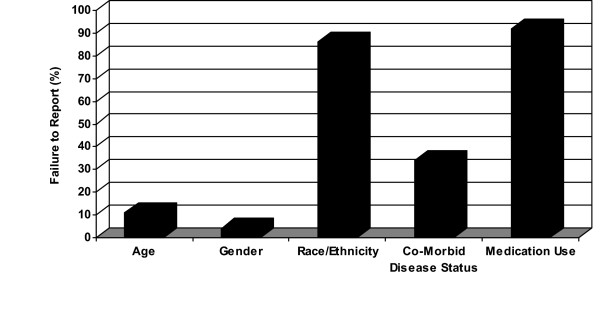
Proportion of selected subject characteristics not reported in randomized trials. Values reported are means. Co-morbid disease status included the reporting of diabetes, cardiac diseases, cancer, hypertension, or any other metabolic disease.

Of the 231 articles that met eligibility criteria for the present study, subjects' age was not reported in 25 (11%) articles. Investigators of ten (4%) different studies failed to report the gender of their study participants. Race and/or ethnicity of study subjects were not reported in 199 (86%) studies. In addition, 78 (34%) studies failed to report the health status (i.e., presence or absence of co-morbidities) of their study subjects at baseline. Further, medication use was not reported in 213 (92%) of the articles (Figure 2). For postmenopausal status, 89 articles were included in the overall analysis with only 7 (8%) studies that did not report the postmenopausal status of their female subjects.

### Sample size and attrition

**Figure 3:**
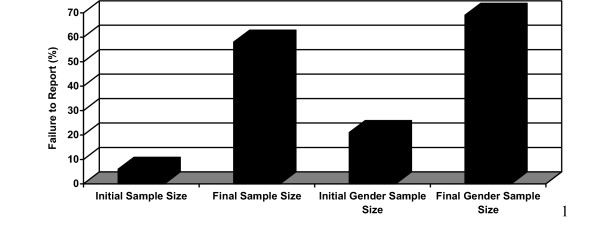
Proportion of articles that did not report initial and final sample size by group or gender in randomized trials. Values reported are means.

Initial sample size was not reported in 14 (6%) studies while final sample size was not reported in 133 (58%) of the 231 eligible articles. When reporting initial and final sample size by gender, 68 articles were included in the analysis. Of the eligible articles, 14 (21%) did not report initial sample size by gender while 47 (69%) failed to report final sample size by gender (Figure 3).

## Discussion

The present study evaluated the quality of reporting of RTs and RCTs in the diet and exercise literature for weight loss, focusing exclusively on subject characteristics, including age, gender, race, health status, medication use, postmenopausal status, and attrition. Transparent reporting of subject data is important in the scientific literature so that readers can efficiently evaluate outcomes in RTs and RCTs. Inadequate reporting creates numerous difficulties with interpretation and can lead to biased conclusions. For example, the effects of diet and/or exercise can vary based upon an individual's age. Schwartz et al. 1991[11] compared the effects of 6 months of endurance exercise (4 days/wk, 45 minutes/day, 85% of heart rate reserve) in older (Mean = 67.5 yrs, SD = 5.8 yrs) versus younger (Mean = 28.2 yrs, SD = 2.4 yrs) on body weight and composition. They found that older men had a 2.5 kg reduction in body weight, a 2.3% decrease in percent body fat, and a 2.4 kg decline in fat mass compared to no changes in the younger individuals. The findings suggest that subjects' age is an important determinant in the response to exercise. However, despite the difference in response to exercise based on the age of subjects, we found that 11% of authors failed to report their subjects' ages within their published report.

Reporting gender of the study participants is also important as men and women respond differently to diet and/or exercise treatments. For example, Donnelly et al. [12] reported that following 16 months of verified, supervised aerobic exercise at 45 minutes per day, 5 days per week resulted in a decrease in body weight of 6% for the men compared to no change in the women.

It also has been shown that individuals from different races and/or ethnic background may respond differently to diet and/or exercise. Jakicic et al. [13] reported that resting energy expenditure was lower in African-American women (Mean = 7279 kJ/d, SD = 825 kJ/d) compared to Caucasian women (Mean = 7807 kJ/d, SD = 854 kJ/d) even after correcting for body weight and lean body mass. The authors concluded that such differences might partially explain the smaller weight losses typically seen in African-American women when compared to Caucasian women enrolled in a weight loss program. In a recent meta-analysis on walking and resting blood pressure in adults, the investigators reported that only 13% of their included studies reported information on race [5]. In the current investigation 86% of authors failed to report the race/ethnicity of the subjects, which highlights the need for caution when extrapolating findings from one population to another.

In the present study, co-morbid health conditions of study subjects were only marginally reported (66%) in the literature. It is well known that certain disease states, such as diabetes and cancer, affect metabolism [14-17], which can influence the effect of diet and exercise on body composition. Closely related to the need for reporting health conditions of study participants is the issue of medication use. Davis et al. [18] determined the effects of taking an antihypertensive medication (i.e., atenolol or chlorthalidone) compared to a placebo group combined with weight loss. They found that at 6 months those taking chlorthalidone lost (6.9 kg) the most weight compared to either the placebo (4.4 kg) or atenolol (3.0 kg) groups. They speculated that the group taking chlorthalidone might have reduced appetite or increased fat mobilization due to the volume depletion and increases in serum and urinary catecholamines. However, despite the profound effects that medication may have on body weight and composition 92% of authors failed to report medication use in our study. Without an adequate description of subject characteristics, readers are unable to judge the comparability of study groups.

Menopause is associated with decreases in lean mass and increases in fat-mass [19-22]. Therefore, post-menopausal status and the associated decline in estrogens, androgens, and other hormones may significantly influence the effects of dietary/exercise interventions on body composition. It appears from the present study the majority of investigators (92%) of dietary/exercise studies report post-menopausal status of their subjects.

Attrition may threaten the internal and external validity of the scientific literature as well as the efficacy of a specific dietary/exercise intervention [23,24]. For example, failure to report the number of dropouts and completers prevents the reader from calculating attrition rates for different experimental conditions, which can result in an overestimate of treatment effectiveness. In the present study, we found that end of study sample size was not reported in the majority (58%) of studies. Without knowledge of the number of subjects who were lost to follow-up, readers are unable to judge the effectiveness of a clinical treatment or ascertain whether or not a research finding has practical significance.

## Conclusion

In the present critical appraisal of the methodological quality of weight loss studies, we found major shortcomings in the reporting of subject characteristics as they relate to enrollment, allocation, and follow-up in trials that evaluated diet and exercise interventions. Many studies did not report variables that may explain some of the variance in outcomes. These findings are consistent with those of similar studies, which indicate inadequate reporting of subject characteristics [5,25] and reveal that poor adherence to published standards of reporting is common [26]. Clearly, additional attention should be paid to ensure compliance with reporting standards for diet and exercise intervention studies.

## List of abbreviations

Consolidation of the Standards of Reporting Trials (CONSORT), randomized controlled trials (RCTs), randomized trials (RTs), kilojoules per day (kJ/d), standard deviation (SD), kilograms (kg), years (yrs)

## Competing interests

The author(s) declare that they have no competing interests.

## Authors' contributions

**CAG **made substantial contributions to the conception and design, acquisition of data, analysis and interpretation of data, and drafting and revising the manuscript for important intellectual content. **EPK, JDL, BWB **and **GH **made substantial contributions to the submitted manuscript by assisting with the acquisition of data, analysis and interpretation of data, drafting of the manuscript, statistical analyses, and critical revisions. **JED **made substantial contributions to the manuscript by helping with the conception and design, critical revisions of the manuscript, and obtaining funding. All authors read and approved the final manuscript.

## Funding source

Supported by National Institutes of Health NIH DK56303, Joseph E. Donnelly, Ed.D., principal investigator

## Pre-publication history

The pre-publication history for this paper can be accessed here:


